# Bioengineered siRNA-Based Nanoplatforms Targeting Molecular Signaling Pathways for the Treatment of Triple Negative Breast Cancer: Preclinical and Clinical Advancements

**DOI:** 10.3390/pharmaceutics12100929

**Published:** 2020-09-29

**Authors:** Dima Hattab, Athirah Bakhtiar

**Affiliations:** 1 School of Pharmacy, University of Jordan, Queen Rania Street, 11942 Amman, Jordan; deemahattab80@yahoo.com; 2 School of Pharmacy, Monash University Malaysia, Bandar Sunway, 47500 Selangor, Malaysia

**Keywords:** triple negative breast cancer (TNBC), RNA interference, small interfering RNA (siRNA), nanotechnology, gene silencing

## Abstract

Triple negative breast cancer (TNBC) is one of the most aggressive types of breast cancer. Owing to the absenteeism of hormonal receptors expressed at the cancerous breast cells, hormonal therapies and other medications targeting human epidermal growth factor receptor 2 (HER2) are ineffective in TNBC patients, making traditional chemotherapeutic agents the only current appropriate regimen. Patients’ predisposition to relapse and metastasis, chemotherapeutics’ cytotoxicity and resistance and poor prognosis of TNBC necessitates researchers to investigate different novel-targeted therapeutics. The role of small interfering RNA (siRNA) in silencing the genes/proteins that are aberrantly overexpressed in carcinoma cells showed great potential as part of TNBC therapeutic regimen. However, targeting specificity, siRNA stability, and delivery efficiency cause challenges in the progression of this application clinically. Nanotechnology was highlighted as a promising approach for encapsulating and transporting siRNA with high efficiency-low toxicity profile. Advances in preclinical and clinical studies utilizing engineered siRNA-loaded nanotherapeutics for treatment of TNBC were discussed. Specific and selective targeting of diverse signaling molecules/pathways at the level of tumor proliferation and cell cycle, tumor invasion and metastasis, angiogenesis and tumor microenvironment, and chemotherapeutics’ resistance demonstrated greater activity via integration of siRNA-complexed nanoparticles.

## 1. Introduction

Cancer is heterogeneous complex disease characterized by rapid and uncontrollable cell proliferation resulting from genetic and epigenetic mutations [1,2]. Cancer burden is greatly significant; it is the second leading cause of morbidity and mortality in the word and is the first in low- and middle-income countries [3,4]. Breast cancer is one of the most common cancers in women [5]. Globally, breast cancer is the leading cause of mortality among females [6]. The overall breast cancer-related mortality rate increased in the past decade, although the overall death rate has declined significantly from 1990 to 2017. The early detection of breast cancer and the advances in cancer treatments and diagnostic tools are the major contributor to decreasing the overall cancer-related mortality [7].

From the molecular level and according to the expression and/or lack expression of hormone receptors (HR); estrogen receptors (ER) and progesterone receptors (PR) and HER-2 on breast cancerous cells, breast cancer is categorized into four subtypes: Luminal A and B characterized by expression of hormonal receptors with or without HER-2, basal-like cancerous cells where the three hormonal receptors are deficient, so they are commonly known as triple negative breast cancer (TNBC), and HER-2 enriched cancer that is enriched with HER-2 only [6,8]. Among the different subtypes of breast cancer, TNBC is clinically deliberated to be the most aggressive form with poorer prognosis that attributes for patients’ death within five years post-diagnosis [9]. TNBC has higher recurrence rate that occur usually within shorter average time [10]. To date, there are no FDA-approved targeted therapies for treating this aggressive form of breast cancer [11]. Patients with TNBC are treated systemically with chemotherapeutic agents which include anthracyclines and taxanes [8,12]. Numerous obstacles compromise the clinical usefulness of these medications, and some of these obstacles are related to the disease itself; as cancerous cells should not only be treated for their uncontrolled cell proliferation, rather integration of cancer metastasis activity, the heterogeneity of the tumor microenvironment, and the complexity of the signaling pathways must be contemplated in choosing appropriate treatment [13]. Other hurdles are associated with the chemotherapeutic agents; lacking selectivity towards cancerous tissues, poor drug delivery, and accumulation leading to dose-limiting and long-term systemic toxicity [14,15,16,17]. Additionally, most cytotoxic agents were designed to destroy the cancerous cells rather than affecting the damaged and/or mutant oncogenes that cause aberrant growth and differentiation of tumor cells [18]. As a result, in most TNBC patients, these agents cause cancer exacerbation, resulting in relapse, cancer metastasis, and therapeutic resistance [19,20]. It is, therefore, a desirable approach to identify new treatment modalities that involves targeted delivery of agents and able to modulate the damaged genes that are involved in cancer pathways.

High diversification of genetic mutations in TNBC patients is the fundamental barrier for effective cancer treatment. Tumor initiation and development is driven by a group of genes that are normally present in healthy cells, in damaged or mutated condition [21]. Overexpression of one or more of these susceptible oncogenes favor uncontrolled cell division, inhibition of the cell growth, and avoidance of the immune system, blockage of cell death, and drug resistance [22]. Targeting suppression of the expressions of oncogenes is one of the paramount important strategies in the treatment of cancer. A number of methods were employed to knockdown the genes that are involved in cancer pathophysiology, but none of them succeeded in complete suppression of the gene [23]. In 1998, Fire and his colleagues revealed that short double-stranded RNA (dsRNA) causes selective suppression of the targeted gene expression. This novel method of controlling the gene expression was termed RNA interference (RNAi) [24]. In RNAi, short double stranded RNAs (dsRNAs) or micro RNAs (miRNAs) is processed into a short interfering RNAs (siRNAs), capable of silencing sequence-specific genes. The siRNA were loaded into the effector complex RNA-induced silencing complex (RISC) to unwound, where single-stranded RNA hybridizes with messenger RNAs (mRNA) target, resulting in gene silencing and down-regulation of certain proteins [25,26,27].

Successful gene silencing necessitates identification of the targeted genes involved in specific cancer signaling pathways and fabrication of the desired sequence of siRNA that is delivered specifically to its site of action in the targeted cells to selectively knockdown the expression of susceptible oncogene. In vivo systemic administration of naked siRNAs is limited due to their physicochemical and pharmacokinetics properties. Naked siRNA has an average diameter of <10 nm, which often undergo rapid renal clearance, causing inefficient accumulation in the targeted tissues. siRNA may be engulfed by tissue monophages, circulating monocytes, and phagocytic cells, which are part of reticuloendothelial systems (RES), to clear foreign bodies and eliminate cellular debris. Naked siRNAs are also vulnerable for biodegradation by serum nucleases and extracellular enzymes Negatively-charged siRNA from its phosphate backbone further prevents efficient anionic cell membrane from cellular internalization. The fate of successfully internalized siRNA were also greatly influenced by cell surface recycling, lysosomal degradation by lysozyme within cytosol and intracellular compartments release. All these criteria mentioned cause systemic delivery of siRNAs deemed challenging [28,29,30]. A growing number of studies have been implicated to develop safe and effective siRNA-targeted delivery systems with the means of utilizing viral and non-viral vehicles [31,32]. The potential immunogenicity, toxicity, poor cell targeting, and expensive production are among the most important challenges that limit the comprehensive application of the engineered viral carriers [33,34]. Alternatively, biocompatible and biodegradable non-viral vectors are promising gene transfer vehicles that shield siRNAs from biological degradation and rapid clearance and circumvent immune system activation. [35]. Diversity in the non-viral-based delivery system has been attributed to remarkable innovations in siRNA-based anti-cancer therapies including nanoparticles.

With the advancement of nanotechnology, nanoparticles are the vehicle of choice for naive siRNA targeted delivery. Nanocarriers have remarkable physicochemical features, including the surface functionality and the particle size that promote their rational development for siRNA delivery [36]. Neutral or negatively-charged nanoparticles are more liable for specific cellular uptake and have longer circulation time. The engineered delivery carriers are often optimized to be in the nanoscale from 5 nm to 100 nm [36,37]. Large nanoparticles with sizes greater than 100 nm are susceptible to non-specific accumulation in healthy organs causing unwanted side effects, whereas small nanoparticles with sizes less than 5 nm have lower circulation half-life as their size are below the renal threshold, so they are prone to renal elimination [37]. Nanoparticles within the range of 5–100 nm prolonged circulation time and reduce protein opsonization [37,38]. There are different studies performed on fabricating nanoparticles using many methods, including co-precipitation, microemulsion, and hydrothermal synthesis [39].

Accumulation of nanoparticles in the targeted tumor cell is enhanced by the permeability and retention effect (PRE), resulting from defective angiogenesis and dysfunctional lymphatic drainage [40,41]. Nanocarriers in the proposed range are passively accumulated in the tumor cells resulting in higher blood residence time, improved drug efficacy, and minimum toxicity [40]. The heightened nanosize is contributed by the penetration and distribution of the nanoparticles within the targeted tumor cell interstitium towards its predetermined site of action [42,43]. Physicochemical properties of nanoparticles were tuned and/or targeting moieties that employ one of the distinctive characteristics of the tumor microenvironment (TME), such as hypoxia, acidosis, or high interstitial fluid pressure (IFP), can be conjugated to the nanoparticles in order to enhance their penetration deeply in the tumor tissues [44,45]. Numerous nanoplatforms were designed to exploit TME characteristics, such as gelatin-coated lipid nanoparticles containing tyrosine kinase inhibitors and PAMAM dendrimer containing tertiary amino group. In the former platform, gelatin is degraded by matrix metalloproteinases (MMT) which is aberrantly upregulated in TME, whereas tyrosine kinase inhibitors suppress *Bcr-Abl* gene and platelet derived growth factor (PDGF) expression, hence reducing IFP [46]. The tertiary amino group in PAMAM dendrimer protonates in acidic media and enhance the drug release and cellular uptake [47]. Xu et al. developed an acidity-sensitive linkage-bridged copolymer of polyethylene glycol (PEG) and poly lactide-co-glycolide (PLGA) encapsulating siRNA, where PEG was seen detached at acidic TME and siRNA was released and internalized into tumor cells [48].

Owing to their diminutive-size, these nano-formulations improve the solubility and serum stability of siRNAs which, consequently, enhance their oral bioavailability, and siRNA nano-encapsulation modifies the pharmacokinetic properties of siRNAs protecting them from serum degradation, renal and hepatic elimination, improving distribution, and targeting siRNAs activity. Additionally, stimuli-mediated nano-therapeutics permits enhanced cellular internalization and intracellular drug release and decreasing the cancerous cells resistance toward siRNAs [49,50]. The most important goals of an ideal nanocarrier are to introduce siRNAs in a safe, biocompatible, biodegradable, and non-immunogenic manner [51,52].

Clinical application of siRNA-based nanotherapeutics for treatment of many health concerns, in general, and breast cancer, in particular (Table 1), poses many advantages. siRNA-based therapies demonstrate remarkable safety-effectiveness profiles: they are non-teratogenic and mutagenic, as they interfere with the late translational stage of the gene expression, and they are highly effective as they suppress targeted genes preferentially and selectively [26,28]. siRNA-based therapeutics are readily fabricated and modified to effectively knock down any gene in the targeted tumor cells with minimal off-target effects and immunogenicity [53]. Several siRNAs can be simultaneously-incorporated within the same carrier, affecting multiple genes’ expressions which, in turn, improves the antitumor effectiveness without increasing cytotoxicity.

## 2. Pre-Clinical Activity of Engineered siRNA-Mediated Therapies for Treatment of TNBC

**Figure 1:**
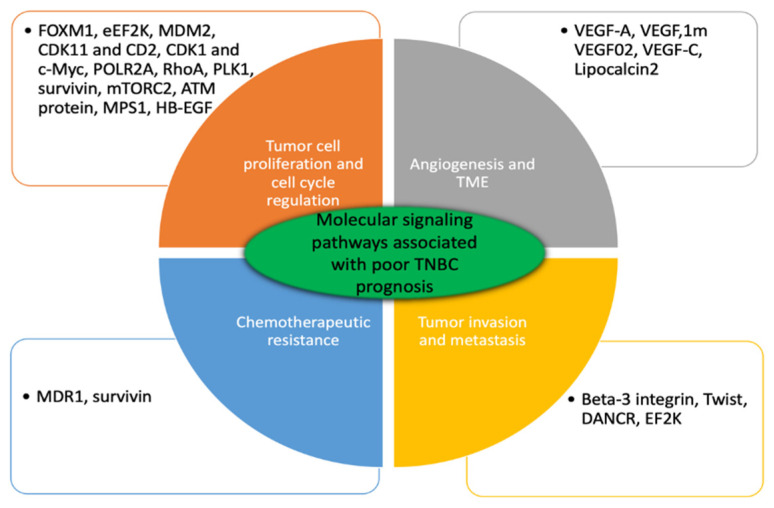
Major genes involved in substantial molecular pathways affiliated with poor TNBC prognosis. These genes are upregulated in patients with TNBC. Bioengineered siRNA mediated anticancer therapeutics targeted single or multiple genes knockdown their suppression and effectively improve disease progression.

TNBC is characterized by inter- and intra-individual diversity of gene mutations. TNBC genes mutations that are concomitant with poor TNBC prognosis are involved in substantial signaling pathways including: cell proliferation and disease progression, cell survival and death, angiogenesis, cancer metastasis and drug resistance (Figure 1). Growing body of evidence revealed promising results concerning the effectiveness of targeted gene suppression using engineered siRNA mediated nanocarriers in the following major areas: Tumor cell proliferation and cell cycle regulation, tumor invasion and metastasis, angiogenesis, and tumor microenvironment and chemotherapeutics resistance (Table 2).

### 2.1. Tumor Cell Proliferation and Cell Cycle Regulation

Forkhead box protein M1 (FOXM1) is a key oncogenic transcription factor, which plays a crucial role in regulating the expression of genes involved in cancerous cell proliferation and cell cycle progression. It was found that FOXM1 is significantly overexpressed in patients with TNBC. Hamurcu et al. examined the effectiveness of liposomal FOXM1 siRNA in mice implanted with MDA-MB-231 cells and found that these gene therapeutics cause a pronounced downregulation of FOXM1 expression and consequently suppress TNBC cells growth significantly [66]. Wang et al. further revealed incorporation of anti FOXM1 siRNA into polyethylimine (PEI)-based cationic polymers enhances cellular uptake of the targeted therapy within the tumor cells for 24 h after its administration. Both in vitro and in vivo studies indicated that PEI-based anti-FOXM1 siRNA knockdown the expression of FOXM1 causing reduction in the protein levels associated with cell proliferation and disease progression [67].

Eukaryotic Elongation Factor 2 Kinase (eEF2K) is a principal regulator of tumor growth and progression. Patients with TNBC had significant elevation in eEF2K expression. As FOXM1 and eEF2K are involved in the same functions in TNBC cells, it was expected that FOXM1 regulates the expression of eEF2K. Hamurcu et al. demonstrated that the engineered siRNA liposomal nanoparticles were able to bind to the promoter region of eEF2K and regulates its expression [66]. Modified gold anti-eEF2K siRNA nanoparticles are highly effective for treating TNBC cells on xenografted mice resulting in a remarkable down-regulation of eEF2K expression and, thus, inhibiting tumor growth and progression [68].

MDM2 is an oncogene that inactivates the tumor suppressor p53, which are greatly overexpressed in TNBC patients. Overexpression of MDM2 leads to suppression of p53 affecting tumor cell proliferation and apoptosis. Single-walled carbon nanotubes (SWNTs) were functionalized with PEG for efficient delivery of siRNA for targeting MDM2. It was concluded that PEG-modified SWNTs are effective drug delivery system promoting major MDM2 silencing effect causing tumor cell proliferation suppression and induced apoptosis in breast cancer cells [69].

Cyclin dependent kinase (CDK) 11 and casein kinase 2 (CK2) are important protein kinases for cancerous cell survival, which are also highly expressed in TNBC cells. Kren et al. prepared novel Tenfibgen-coated nanocapsules (TBG nanocapsules) containing anti-CDK11 siRNA or anti-CK2 siRNA targeting Tenascein-C receptors that are found abundantly within the breast cancer stroma. Intravenous administration of these gene therapeutics suppresses mRNA and protein expression causing significant reduction in tumor cell proliferation and growth [70].

Several studies demonstrating molecular targeting of v-Myc myelocytomatosis viral oncogene homolog (c-Myc) that is highly expressed in TNBC. The significance of c-Myc overexpression arises when its suppression is concomitantly associated with inhibition of CDK1 expression. Downregulation of these two genes results in specific targeting and synthetic lethality in TNBC cells. As c-Myc, CDK1 plays a key role in cell death and apoptosis. Administration of anti-c-Myc siRNA-encapsulated nanoparticles causes significant c-Myc gene silencing that contributes to a slight therapeutic effect on TNBC cells whereas the delivery of siRNA targeting CDK1 results in silencing of CDK1 expression only in TNBC cells containing high levels of c-Myc expression. The combination is ideal not only for its pronounced decreased cell proliferation and cell apoptosis, but also for its selective and specific targeting of TNBC cells with no adverse events on normal breast cells [71].

Tumor suppression (*TP*53) mutation is one of the most frequent oncogenic mutations in TNBC. Remarkable preclinical studies were conducted to reestablish *TP*53’s effect but none of them were transformed into human clinical studies. Hemizygous loss of *TP*53 is the most recurrent TNBC mediated *TP*53 mutations and is associated with deletion of neighboring genes, most importantly, hemizygous deletion of *POLR2A* [69]. Antitumor-based silencing of *POLR2A* expression was investigated by Xu and his coworkers. A pH-sensitive nano-bomb was developed incorporating antiPOL siRNA and guanidine-CO_2_ functionalized chitosan (CG) at neutral pH. siRNA-nanoplatforms-containing CG-CO_2_ were not only efficaciously internalized into TNBC cancerous cells harboring hemizygous *TP*53 deletion, but also able to escape the intracellular trafficking and release siRNA at low pH. TNBC cell lines embracing hemizygous loss of *POLR2A* are more sensitive than those harboring the wild type for siRNA nanotherapeutics, as a result, knockdown of *POLR2A* expression cause selective TNBC cell death with no cytotoxicity. Inhibition of tumor growth by targeting *POLR2A* was not influenced with the status of *TP*53 loss [70].

Ras homologous A (RhoA) and Ras homologous C (RhoC) are low molecular weight GTPases of the Ras family. Significant high levels of RhoA and RhoC are reported in all subtypes of breast cancer especially TNBC. Overexpression of RhoA enhances tumor cell proliferation, and induces angiogenesis and tumor invasion and metastasis [72]. Li et al. studied the antitumor effect of anti-RhoA siRNA nanoparticles in vivo where chitosan-coated polyisohexylcyanoacrylate (PIHCA) nanoparticles encapsulating anti-RhoA siRNA were fabricated and given to MDA-MB-231 xenografted mice intravenously. Complete suppression and more than 90% inhibition of tumor growth were perceived after intravenous administration of anti-RhoA siRNA [73]. As RhoA and RhoC are expressed exclusively in cancerous cells only, specific and selective targeting of RhoA is a promising candidate for treatment patients with TNBC [72].

Polo-like kinase 1 (*PLK1*) is extensively upregulated in patients with either primary or metastatic TNBC. Inhibition of *PLK1* expression is associated with ameliorating tumor cell death in a wide variety of metastatic cancer [74,75]. Targeted nanoplatforms incorporating anti-*PLK1* siRNA encapsulated within antibody-conjugated bioreducible cross-linked with PEI and polyethylene glycol (PEG) layer-by-layer with coated mesoporous silica nanoparticles (MSNP) showed the following characteristics: PEI is highly effective for siRNA binding and escapes the intracellular trafficking. PEG is incorporated for siRNA protection by enzymatic degradation, reducing PEI toxicity, circumventing the immune system, and decreasing nanoparticles’ aggregation. In vitro and in vivo studies revealed that these nanoparticles were efficiently internalized into targeted TNBC metastatic cells overwhelming both cancer hallmarks: tumor cell proliferation and tumor metastasis. It is worth noting that reactive oxygen species’ (ROS) scavenging ability of MSNP contributes to the antimetastatic effect, whereas the internalized anti-*PLK1* siRNA exhibits apoptotic cellular death activity [76].

MDM2 [77], cyclin-dependent kinase (CDK) 11 and casein kinase 2 (CK2) [78], c-Myc [79], survivin (also known as BIRC-5, an inhibitor of apoptosis protein) [80,81], mTORC 2 [82], Ataxia-telangiectasia mutated (ATM) protein [83], monopolar spindle 1 (MPS1, most commonly known TTK protein kinase) and cell division cycle protein 20 (CDC20) [84], Heparin-binding EGF-like growth factor (HB-EGF) [85], epidermal growth factor receptors (EGFR) [86,87,88], CXCR-4 [89], luciferase mRNA protein [90], and enhanced green fluorescent protein (eGFP) [91] are overexpressed in TNBC cells. Gene function studies showed that silencing any of these molecular factors using siRNA-mediated nanoparticles caused significant tumor growth suppression. Many other genes, such as the brother of the regulators of imprinted sites (BORIS) [92], *HER2/neu* gene [93], *E2F3* [94], and *Akt 1*, *2*, and *3* [95], are involved in tumor cell cycle regulation and cell proliferation. Pre-clinical in vitro studies confirmed that downregulation any of these genes’ expression using siRNA-based gene therapy showed encouraging results in inhibition tumor cell proliferation and cell survival.

### 2.2. Tumor Invasiveness and Metastasis

Tumor metastasis is the primary factor associated with cancer related deaths in patients with TNBC. Epithelial mesenchymal transition (EMT) is the driving force for tumor invasiveness, metastasis, drug resistance, and remodeling of TME [96]. The morphological alterations of the normal epithelial cells’ characteristics into the mesenchymal phenotype allow the cancerous cells to disseminate out into distant organs [97,98]. Central nervous system metastasis and lung metastasis are among the utmost fatal metastatic repercussions in patients with TNBC [20]. Upregulation of numerous transcriptional factors is among the foremost causes to induce EMT. Epidermal growth factor (EGF), fibroblast growth factor, hepatocyte growth factor, transforming growth factor (TGF)-β, and bone morphogenetic proteins are EMT master regulators that govern the development and progression of breast cancer metastasis [99,100].

Breast cancer metastasis and EMT are governed by the transmembrane protein β3 integrin. Upregulation of TGF-β in cancerous cells persuades overexpression of β3 integrin, therefore, it is expected that silencing β3 integrin expression inhibits TGF-β induced EMT, tumor invasion, and metastasis [101]. Parvani et al. prepared a cationic lipid nanocarrier (ECO) containing siRNA that are delivered specifically to the targeted β3 integrin gene. Treatment of TGF-β pre-stimulated TNBC cells with ECO/β3-siRNA nanoparticles reduces TGF-β-mediated EMT, tumor proliferation, and invasion as these engineered nanoparticles prompt inhibition of more than 70% of β3 integrin expression for approximately seven days. Downregulation of mesenchymal markers (N-cad and PAI-1) and upregulation of epithelial markers (E-cad and CK-19) attributes to inhibition of TGF-β induced EMT. Cellular uptake of β3-siRNA based nanoparticles into targeted cells was improved by surface modifications of the engineered nanoparticles with RGD peptide. In vivo analysis of the modified ECO/β3-siRNA nanoparticles decreased the primary tumor burden and tumor recurrence, designating that systemic administration of modified siRNA nanoparticles demonstrated significant suppression of tumor metastasis and invasion [102].

Patients with TNBC and ovarian cancers are characterized by elevated levels of TWIST. TWIST is a major transcription factor activates EMT, promotes stem like phenotyping, and inhibits apoptosis, increasing the risk of tumor recurrence and poor prognosis [103]. Inhibition of TWIST overexpression in TNBC cells was examined by the administration of poly(amidoamine) dendrimer (PAMAM) loaded with TWIST-siRNA. The modified siRNA-based dendrimers were efficiently internalized into TNBC cells causing significant reduction in TWIST expression accompanied by phenotyping changes associated with decreasing cancerous cells motility, decreasing TWIST-mediated upregulation mesenchymal markers (Vimentin and N-cad) and attenuating the dissemination and invasion of TNBC cells for approximately one week after dendrimer treatment. As TWIST protein is expressed only in cancerous cells and not in normal breast cells, silencing TWIST expression using siRNA-based dendrimer is a promising safe and biocompatible approach for treating breast cancer-associated metastasis [104].

A novel class of RNA consisting of more than 200 nucleotides, so-called long noncoding RNAs (lncRNAs), are vital regulators of gene expression associated with cellular differentiation and development [105]. The oncogenic lncRNAs have a pivotal role in inducing malignant cell proliferation, invasion, and metastasis [106]. BMP/OP-Responsive Gene (*BORG*) [107], HOX Antisense intergenic RNA (*HOTAIR*) [108] and Differentiation Antagonizing Non-Coding RNA (*DANCR*) [109] are lncRNAs concerned in tumor recurrence and progression, indicating poor cancer prognosis. As *DANCR* is highly expressed in TNBC cells, Vaidya et al. revealed targeting *DANCR* lncRNAs causes suppression of TNBC metastasis, and inhibition of cell proliferation and survival. Vaidya and his colleagues prepared RGD-PEG-ECO nanoparticles loaded with anti-*DANCR* siRNA. In vitro transfection of MDA-MB-231 and BT549 cells with anti-*DANCR* siRNA showed significant reduction of cancer cell proliferation and inhibition of tumor cell migration and invasion. Targeting *DANCR* in MDA-MB-231 xenografted mice using RGD-PEG-ECO/siRNA-engineered nanoparticles results in decreasing tumor volume and the suppression of tumor growth [109].

A recent study examined the silencing effect of EF2 kinase in tumor proliferation, apoptosis, invasion, and metastasis. EF2K is a typical kinase that is overexpressed in *BRCA1*-mutated breast cancer cells. Silencing EF2K was performed by treatment of HCC-1937 and MDA-MB-436 cells with cobalt-ferric anti-EF2K siRNA nanoparticles where internalization of anti-EF2k-mediated siRNA into TNBC cells were enhanced in comparison to normal breast cells. Cobalt-ferric anti-EF2K siRNA nanoparticles knocked-down the expression of EF2K in transfected TNBC cells and, hence, inhibited TNBC cells proliferation and colony formation and impaired cell migration and invasion. Targeting of EF2K in *BRCA1*-mutated MDA-MB-436 and HCC-1937 cell-xenografted mice inhibited tumor growth, reduced cancer cell proliferation, angiogenesis, and induced apoptosis. Remarkably, COFe-siRNA nanoparticles showed no obvious cytotoxicity [110].

### 2.3. Angiogenesis and Tumor Microenvironment

Both tumor growth and metastasis are driven by angiogenesis [111]. Tumor angiogenesis is characterized by high neovascular density, high lymphatic density, and vascular invasion. Numerous angiogenic factors are secreted in the tumor microenvironment stimulating new blood vessels sprout. Angiogenic factors overexpression is correlated with cancer development as vascular endothelial growth factors (VEGF) especially VEGF-A subtype, attributed for cancer progression such insulin like growth factors (IGF) and TGF-β1 and concerned with tumor invasion and metastasis like vascular endothelial growth factor receptors (VEGFRs) [112]. These proangiogenic factors are more pronounced in TNBC compared to other subtypes of breast cancer [111,113].

It was reported that inhibition of VEGF using monoclonal antibodies is not effective for treating patients with TNBC as theses anti-VEGF antibodies hinder VEGF interaction with their receptors at the endothelial cells and the plasma membrane of the tumor cells without affecting the internal VEGFR expressed at the nuclear envelope [114,115,116,117,118,119,120,121,122]. Salva et al. prepared chitosan nanoplexes encapsulating siRNA targeting VEGF-A, VEGFR-1, VEGFR-2, and neuropilin-1 (co-receptors for VEGF, NPR-1). Intratumoral administration of VEGF/siRNA nanoplexes into TNBC induced rats declined VEGF expression by more than 80% and reduced the tumor volume by more than 90%. Chitosan/siVEGF-mediated nanoplexes transfection did not increase interferon mediated immune response. Salve et al. demonstrated the silencing effect of the combination of VEGF genes cause potent and specific inhibition of breast cancer growth [117]. In another study, PLEGP nanocomplex containing anti-VEGF siRNA was developed. Different PLEGP nanocomplexes were investigated for their anti-tumor and cytotoxic activity. PLGEP_1800_ was shown to have a modest downregulation of VEGF expression with no significant cytotoxicity. In vitro studies revealed that transfection of MDA-MB-231 cells with PLGEP_1800_ nanocomplex suppressed cancerous cell migration and invasion. Vascular endothelial cell proliferation and tube formation were repressed in PLEGP_1800_/VEGF siRNA transfected HUVECs cells. Knock down of VEGF expression and subsequent inhibition of CD31 level in siVEGF treated MDA-MB-231 xenografted mice indicated suppression of tumor growth and impairment of angiogenesis [118].

Many identifiable factors attribute for lymphangiogenesis in breast cancer. Among them is VEGF-C that activates the lymph vascular cells and lymphatic cells growth. Upregulation of VEGF-C level indicates tumor metastasis particularly for the lymph nodes. The silencing of VEGF-C expression was studied by Chen and his colleagues using siRNA mammalian vectors. Anti-VEGF-C siRNA knocked down VEGF-C mRNA and VEGF-C protein production in C166 cells. Downregulation of VEGF-C expression leads to suppression lymphangiogenesis but not angiogenesis, suppression of regional and distant metastasis implicated by inhibition of lymph node metastasis and spontaneous lung metastasis, immunomodulating effect, and improving mice survival [119].

Lipocalin 2 (Lcn2), a subtype of lipocalin protein family, is upregulated in TNBC contributing to tumor progression, invasion and metastasis through its interaction with matrix metalloproteinase-9 (MMP-9) [119]. Lipocalin 2 play a striking role in regulating of angiogenesis, its overexpression in TNBC is accompanied by increased VEGF expression. It is suggested that Lcn 2 induced VEGF production through the Erk/HIF-1α signaling pathway [120]. Elevated urinary levels of Lcn 2 in patients with TNBC correlated with its poor progression representing that Lcn 2 is biomarker for both diagnosis and prognosis of breast cancer [119]. Anti-angiogenic engineered liposomes encapsulating both ICAM-1 and Lcn2 siRNA were prepared (ICAM-Lcn-LP). ICAM-Lcn-LP exposed MDA-MB-231 cells suppress Lcn expression significantly with no cytotoxicity. Ablation of Lnc 2 expression in ICAM-Lcn-LP transfected MDA-MB-231 harvested conditioned media knocked down VEGF expression in MDA-MB-231 cells. ICAM-Lcn-LP transfected MDA-MB-231 harvested conditioned media was co-cultured with HUVEC and HMVEC cells. Silencing of Lcn2 production minimized endothelial cells migration and tube formation in HUVEC and HMVEC cells. Inhibitory effect of TNBC-induced angiogenesis were seen both in vitro and in vivo [121].

Angiogenesis is a key driver for tumor growth and metastasis. Selective targeting, accessibility and low drug resistance liability are major advantages for targeting vascular endothelial cells in addition to cancerous cells [122]. Receptor mediated endocytosis is an innovative tactic targeting receptors available at many cell populations. Nucleolin is a receptor that is highly expressed at both cancerous cells and endothelial cells originating from blood vessels [91]. Novel fabrication of nucleolin targeted siRNA based nanoparticles is a promising approach employing gene delivery for dual therapy. Gomes-da-Silva et al. developed F3 targeted liposomes incorporating enhanced green fluorescent protein (eGFP) and transfected them into MDA-MB-231 cells and HMEC-1 cells. It was reported that F3 targeted liposomes enhanced the cellular uptake by both cancerous cells and endothelial cells but not by non-cancerous cells. Additionally, improved targeted siRNA liposomes internalization by MDA-MB-231 and HMEC-1 cells was accompanied by knock down eGFP expression in both cells [91].

Most TNBC cells exhibits mutation in the tumor suppressor p53 gene. p53 is an oncogene that is overexpressed in patients with TNBC and involved in multiple molecular pathways including tumorigenesis, apoptosis, metastasis and angiogenesis [123,124]. Braicu et al. demonstrated anti-angiogenic impact of silencing mutated p53 gene using p53-siRNA based approach where transfection of Hs578T cells with anti-p53 siRNA induced apoptosis, decreased cell survival and reduced angiogenesis. Treatment of TNBC cells with a combination of p53 siRNA and epigallocatechingallate (EGCG) demonstrated more pronounced anti-apoptotic and anti-angiogenic as compared for each treatment alone [125].

cAMP and its two receptors protein kinase A (PKA) cAMP dependent and exchange protein activated cAMP (EPAC1). EPAC1 is a cAMP activated guanine nucleotide exchange factor. cAMP and its receptors are exposed to be overexpressed in patients with breast cancer; associated with cell differentiation, cell migration and cell survival and death [126]. Cell culture studies revealed that suppression of EPAC1 expression in anti-cAMP siRNA treated MDA-MB-231 cells co-cultured HUVEC cells exhibited activities include cessation in the formation of the nodes and tubes in HUVEC cells, upregulation of the anti-angiogenic proteins involved in adhesion and angiogenesis, downregulation of pro-angiogenic proteins such as fibroblast growth factor (FGF), transforming growth factors (TGF) and many others attributed to cell migration and locomotion, inhibited vascular cells invasion mediated TNBC cells and downregulation of Paxillin and MENA molecules that are concerned with focal adhesion. It is suggested that EPAC1 have a remarkable role in regulating tumor microenvironment, neovascularization and invasiveness of TNBC cells contributing to vascular metastasis [127].

### 2.4. Chemotherapeutic Resistance

Cancerous breast cells are highly sensitive to chemotherapeutic agents with a response rate extent approximately 80% but unfortunately most of the cancerous cells develop resistance contributing to treatment failure and poor TNBC prognosis [128]. Emerged chemotherapeutics’ resistance is incongruent with the type; structure and the biological activity of the chemotherapeutics therefore substitution between different agents is no longer effective [129,130].

**Figure 2:**
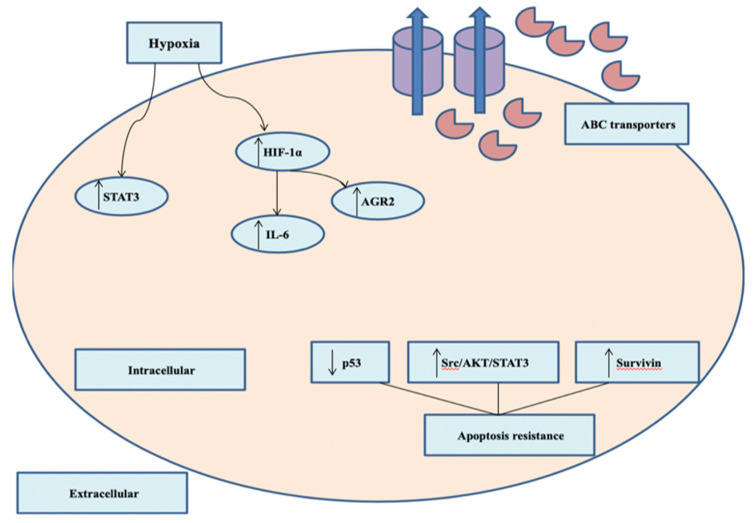
Major mechanisms of chemotherapeutics resistance in TNBC. Overexpression of ABC transporters and extruding chemotherapeutic agents out of the cancerous cells. Hypoxia induces upregulation of HIF-1α and other signaling factors.

Though 90% of the initially chemo-responsive TNBC cells develop resistance, advanced research exploring the impact of siRNA-based nanotherapeutics as a targeted platform to overcome doxorubicin-based [131,132,133] and taxane-based resistance [134,135,136] exploiting non-TNBC cell lines, most commonly MCF-7 cells. Extensive research are exploring the mechanisms of multidrug resistance (MDR) evolved in breast cancerous cells including ATP-binding cassette (ABC) transporter, activation of anti-apoptotic pathways, enhancement chemotherapeutics detoxification, overexpression of β-tubulin III, KIF14, and many other signaling factors, alterations of DNA repair enzymes, and chemoresistance-induced hypoxia and NF-κB signaling pathway [137]. Among these different mechanisms, ABC transporter, alterations of the antiapoptotic genes and the role of hypoxia are major mechanisms of chemoresistance (Figure 2).

Extruding the internalized chemotherapeutics out of the cancerous cells is governed by ATP-binding cassette (ABC) transporter family. Two efflux pump members namely ABCB1 (P-glycoprotein) and ABCG2 (BCRP) transporters are identified [130]. ABCB1 and ABCG2 transporters are overexpressed in patients with TNBC [138]. Clinical studies failed to verify MDR phenotype modulated therapeutics either for their cytotoxicity or for their unwanted pharmacokinetics interactions, thus silencing gene expression mediating thwarting the synthesis of ABC transporters is the most accessible approach to circumvent MDR clinically [139,140]. It was earlier reported that multidrug resistance genes (lung resistance-related protein (LRP), multidrug resistance associated proteins 1 and 2 (MRP1, MRP2), multidrug resistance gene 1 (*MDR1*), and breast cancer associated proteins (BCRP)) mediated mRNA expressions in metastatic breast tumor cells specimens of patients receiving first line chemotherapy based regimens correlates inversely with treatment efficacious with elevated genes expression predicting poor prognosis in those patients [141].

Growing bodies of evidence revealed that knockdown of ABCB1 and ABCG2 expression mediated siRNA-based nanoparticles increased intracellular concentration and cancerous cells chemosensitivity of many cytotoxic drugs such as doxorubicin and paclitaxel [142,143,144]. Deng et al. prepared multi-layers nanoparticles constituting of doxorubicin-based nanoparticles encircled with alternating layers of siRNA targeted *MDR1* and poly-l-arginine enclosed by an outer shell of hyaluronic acid (HA). The engineered drug delivery platform posed high siRNA loading capacity, stability and cellular internalization with high efficacy-toxicity profile. Administration of doxorubicin-siRNA nanoparticle complexes decreases the tumor volume significantly in comparison to the treatment with either anti-*MDR1* siRNA or doxorubicin alone [145].

**Figure 3:**
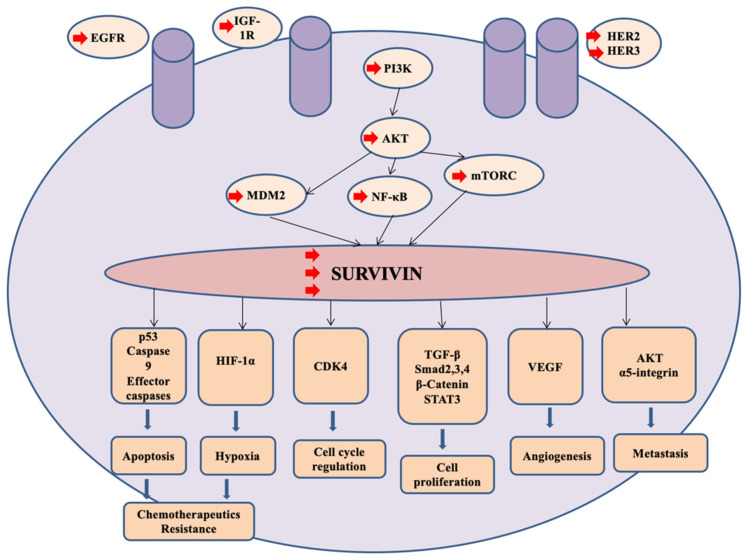
Survivin is a potential target for treatment patients with TNBC. Activation of several kinases such as HER2, HER3, IGF-1R and EGFR elicits PI3K/AKT signaling pathways resulting in upregulation of survivin expression. Overexpression of survivin stimulates numerous genes and proteins associated with cancerous cell proliferation and survival, migration, angiogenesis, and promotes chemoresistance.

Targeting the anti-apoptotic genes/proteins is another major mechanism-induced chemoresistance. Survivin is one of the most attractive inhibitors of apoptosis (IOP) controlling tumor growth, angiogenesis, apoptosis, and recently drug resistance, in particular, paclitaxel (Figure 3) [146]. Therapeutic effectiveness of survivin to enhance TNBC cells sensitivity to chemotherapeutics agents were investigated using polymeric micelles of PEG_2000_-PE incorporating survivin siRNA. Treatment of anti-survivin siRNA PM transfected MDA-MB-231 cells with paclitaxel showed that knockdown survivin expression in resistant cancerous cell enhance paclitaxel cytotoxicity significantly and cause destabilization of microtubules. In addition, co-encapsulation of paclitaxel and anti-survivin siRNA into PM exhibited a significant suppression of tumor growth in comparison to the paclitaxel free anti-survivin siRNA PM [147]. Triple therapies of chemotherapy, gene therapy, and photothermal therapy revealed outstanding antitumor effect in vitro and in vivo in comparison to mono or dual therapies [148].

Chemoresistance-induced kinases in TNBC were identified by screening a kinome of siRNA into doxorubicin treated/untreated MDA-MB-231 cells. siRNA transfected MDA-MB-231 cells showed many potential kinases of doxorubicin-based resistance in TNBC cells. Src kinase is the most significant kinase for doxorubicin-induced chemoresistance not only in TNBC cell lines (MDA-MB-231, MDA-MB-468 and Hs578T) but also in non-TNBC cells (MCF-7 and T47D). Src kinase was highly expressed in TNBC cells rather than in normal breast cells. Src silenced MDA-MB-231 cells showed low protein levels of STAT3 and AKT proposing that Src/AKT/STAT3 signaling pathway play a role in chemoresistance [149]. These results are consistent with Moreira et al. findings that confirmed overexpression of STAT3 protein in doxorubicin-treated TNBC BT-549 cells suggesting that STAT3 is an essential EGF gene involved in doxorubicin-based resistance [150].

## 3. Clinical Trials of Anticancer siRNA-Mediated Nanotherapeutics

**Figure 4:**
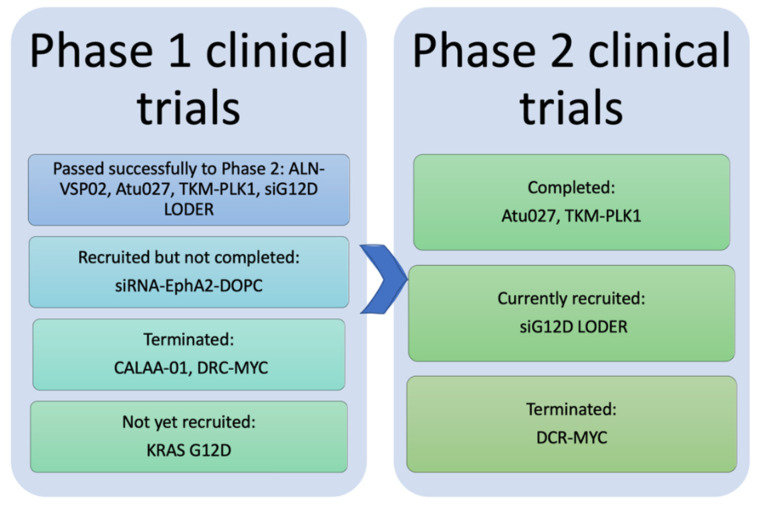
Representative diagram for the results of phase I and phase II clinical trials of the eight anticancer nano-siRNAs. Phase I trials of CALAA-01 and DCR-MYC were terminated. The phase I trial of siRNA-EphA2-DOPC has recruited but not completed yet, whereas the mesenchymal stem cell-derived exosomes with KRAS G12D trial has not yet recruited. ALN-VSP02, Atu027, TKM-PLK1, and siG12D LODER passed phase I successfully. Four siRNA nanotherapeutics moved to phase II; Atu027 and TKM-PLK1 completed phase II while siG12D LODER is currently being recruited. The phase II trial of DCR-MYC was terminated.

Current pre-clinical advances exploiting siRNA-based nanotherapeutics for treatment of cancer, in general, and TNBC, in particular, established the effectiveness of these therapeutics in inhibition cell proliferation and tumor growth, suppression tumor invasion and metastasis, pronounced anti-angiogenic effect, and enhancement of chemotherapeutics efficacy. Remarkably, several siRNA nanoparticles succeeded in being investigated in clinical trials. To date, eight siRNA-mediated nanoparticles have commenced phase I and II clinical trials (Figure 4) for treatment of a wide variety of solid cancers that could be employed for patients with TNBC (Table 3).

EphA2-siRNA-DOPC is the most recent anticancer siRNA-mediated nanotherapeutics investigated clinically. EphA2 is a protein member of tyrosine kinases receptors that are expressed abundantly in the embryos and expressed to a lesser extent in adults mainly at the surface of epithelial cells. Several studies reported that EphA2 is selectively upregulated in diverse arrays of cancer including, but not limited to, breast, pancreas, prostate, lung, and most importantly ovarian cancer affecting cancerous cells growth, invasion, and metastasis and angiogenesis and increasing the tumor burden. EphA2-siRNA was incorporated into liposomal nanoparticles (DOPC) called EPHARNA (EphA2-siRNA-DOPC) specifically targeting EphA2 expression in the epithelial mediated tumor [164,165,166]. In vitro and in vivo studies demonstrated that EPHARNA has anti-angiogenic effect and reduce tumor growth dramatically. Moreover, remarkable tumor growth inhibition was found with simultaneous administration of EphA2-siRNA-DOPC and paclitaxel [167]. In vivo toxicological studies showed that DOPC nanoliposomes exhibit no observed adverse events at dose range of 75–225 mcg/kg after single administration and twice administration results in no major toxicities [168]. In 2015, EphA2-siRNA-DOPC entered phase I trial recruiting patients with advanced metastatic solid cancer receiving intravenous EPHARNA twice weekly over two hours [169].

## 4. Conclusions

Despite comprehensive knowledge and advanced research on breast carcinoma, the cancer burden remains significantly high. High diversification of genomic transmutations and enormous phenotypic alterations attributed to traditional anticancer therapeutics’ failure, which contributes to relapse, recurrence, and cancer-related mortality, especially on TNBC. Most patients with TNBC exhibit drug resistance, invasion, and metastasis causing poor disease prognosis and poor overall patient survival rate. Inter- and intra-individual heterogeneity in TNBC limits intervention and efficacious treatment for TNBC. RNAi interference and, in particular, siRNA-based therapeutics technology attracted attention via salient physicochemical characteristics, and pharmacokinetics properties along with the systemic biodegradation liability mitigate the systemic administration of naked siRNA. Targeted delivery systems are needed to overcome the aforementioned obstacles attaining specific and selective gene-based therapeutic products’ internalization in the tumor cells, sufficient intracellular retention, and efficacious antitumor activity with no off-target side effects. Nanosized platforms aid in encapsulating siRNA protecting it from the external environment, targeting the tumor cells and evading the immune-stimulating effect. Most importantly, multiple drug modalities (chemotherapeutics, photothermal agent and siRNA-based therapeutics) may be incorporated in these nano-formulations. Integrations of different drug modalities in one nanocarrier posed significant antitumor effectiveness compared to the delivery of one agent or sequential administration of multiple agents. Several anticancer-based gene nanotherapies have commenced phase I and II clinical trials and have shown promising results. To date, there are no siRNA-anticancer therapies on the market, but the progression of siRNA-based therapy from the bench to the clinic will open the horizon for these advanced anticancer therapeutics to evolve.

## Figures and Tables

**Table 1:** Effectiveness of siRNA-loaded nanotherapeutics in patients with HR and/or HER2 positive breast cancer.

Breast Cancer Subtype	Cell Line	Target (Gene/Protein)	Type of Nanoparticles	Antitumor Effect	References
HR positive	MCF-7	BCL-2 and BCL-XL	Calcium phosphate pEG-polyanion	Inhibit apoptosis	[54]
		EGFR1 and ERBB2	Carbonate apatite	Inhibit tumor cells proliferation	[55]
		ER, BCL-2, ERBB2, and EGFR	Carbonate apatite	Induce cell death	[56]
		c-*ROS1*	Carbonate apatite	Decrease chemotherapeutic resistance	[57]
		EpCAM	Aptamer polyethylineimine	Inhibit tumor cell proliferation	[58]
		VEGF	PLCP	Inhibit angiogenesis and reduce tumor growth	[59]
		CDK8	Lipofectamine 2000	Inhibit tumor cells proliferation	[55]
		IKKε	Lipofectamine 2000	Reduce tumor invasiveness and inhibit tumor proliferation	[60]
		MAPK	Barium salts nanoparticles	Inhibit tumor growth	[61]
	T-47D	Cyclin E	Oligofectamine	Induce apoptosis	[62]
HR and HER2 positive	BT-474	*HER2*	Mesoporous silica nanoparticles	Improve chemotherapeutics sensitivity	[63]
		*PLK1*	Fusion protein of single chain fragmented antibodies	Inhibit tumor cells growth and metastasis	[64]
Only HER2 positive	SkBr3	IKKε	Lipofectamine 2000	Reduce tumor invasiveness and inhibit tumor proliferation	[60]
		Cyclin E	Oligofectamine	Induce apoptosis	[62]
	HCC1954	*HER2*	Superparamagnetic Iron oxide nanoparticles	-	[65]

**Table 2:** Preclinical studies of siRNA-based therapies mediated antitumor effect.

Target (Gene/Protein)	Type of Nanoparticles	In vitro and/or in Vivo Evaluation/TNBC Cell Line	Reference
**Cell Proliferation and Cycle Cell Progression**			
FOXM1	Liposomal nanoparticles	In vitro and in vivo/MDA-MB-231 cells	[66]
FOXM1	PEI-based cationic polymer	In vitro and in vivo/MDA-MB-231 cells	[67]
eEF2K	PEI-modified gold nanoparticles	In vitro and in vivo/MDA-MB-436 cells	[68]
*POLR2A*	Agarose gel nanoparticles	In vitro and in vivo/MDA-MB-231 and MDA-MB-453 cells	[70]
RhoA	Chitosan-coated polyisohexylcyanoacrylate (PIHCA) nanoparticles	In vivo/MDA-MB-231 cells	[73]
*PLK1*	Mesoporous silica nanoparticles	In vitro and in vivo/BT549 cells and MDA-MB-231 cells	[76]
MDM2	PEG-functionalized SWNTs	In vitro and in vivo/Breast cancer B-Cap-37	[77]
CDK11 and CK2	TBG nanocapsules	In vitro and in vivo/MDA-MB-231 cells	[78]
CDK1 and c-Myc	PEG-PLA nanoparticles	In vitro and in vivo/SUM149 and BT549 cells	[79]
Survivin	Lipid substituted polymer	In vitro/MDA-MB-231 cells	[80]
mTORC2	si-nanoparticles	In vitro and in vivo/BT474 cells, MDA-MB-361 cells, SKBR3 cells and MDA-MB-231 cells	[82]
ATM protein	Nanoliposomes	In vitro and in vivo/MDA-MB-231 cells and SK-BR-3 cells	[83]
MPS1 or TTK	PEI substituted with linoleic acid	In vitro/MDA-MB-231 cells	[84]
HB-EGF	Fab’s antibody modified LNP	In vitro and in vivo/MDA-MB-231 cells	[85]
EGFR	CPP loaded nanobubbles	In vitro and in vivo/MDA-MB-231 cells	[86]
EGFR	Fab conjugated liposomal nanoparticles	In vitro and in vivo/MDA-MB-231 cells	[87]
EGFR	PMLA-based nanobioconjugate	In vitro and in vivo/MDA-MB-468 cells	[88]
CXCR4	Plerixafor-modified nanocarriers	In vitro/MDA-MB-231 cells	[89]
Luciferase mRNA	Cationic nanoparticles	In vitro and in vivo/MDA-MB-435/LCC6	[90]
eGFP	F3-targeted liposomal nanoparticles	In vitro and in vivo/MDA-MB-231 and MDA-MB-435 cells	[91]
**Tumor Invasion and Metastasis**			
β3 integrin	Cationic lipid nanocarrier (ECO), the modified RGD-ECO nanoparticles	In vitro and in vivo/MDA-MB-231 cells	[102]
*TWIST*	PAMAM dendrimer nanoparticles	In vitro and in vivo/SUM 1315 breast cancer cell	[104]
*DANCR*	RGD-PEG-ECO nanoparticles	In vitro and in vivo/MDA-MB-231 and BT549 cells	[109]
EF2K	CoFe-nanoparticles	In vitro and in vivo/MDA-MB-436 and HCC-1937 cells	[110]
**Angiogenesis and Tumor Microenvironment**			
VEGF-A, VEGFR-1, VEGFR-2 and neuropilin-1	Chitosan nanoplexes	In vivo/Breast tumor induced rats	[117]
VEGF	PLEGP_1800_ nanocomplex	In vitro and in vivo/MDA-MB-231 cells, MCF10A cells and HUVEC cells	[118]
Lipocalin 2	ICAM-1 conjugated liposomes	In vitro and in vivo/MDA-MB-231 cells, HUVEC and HMVEC cells	[121]
VEGF-C	Plasmid vector	In vitro and in vivo/C166 cells	[123]
eGFP	F3-targeted liposomes	In vitro/MDA-MB-231 cells, MDA-MB-435S cells and HMEC-1 cells	[91]
**Chemotherapeutics’ Resistance**			
*MDR1*	Layer by layer nanoparticles	In vitro and in vivo/MDA-MB-468 cells	[145]
Survivin	PEG_2000_-PE PM	In vitro and in vivo/MDA-MB-231 and paclitaxel resistant SKOV3 cells	[147]
Survivin	Nanocopolymer	In vitro and in vivo/MDA-MB-231 human breast cells	[148]

Abbreviations: ATM: Ataxia-telangiectasia mutated protein; CDC20: Cell division cycle protein 20; CDK: Cyclin dependent kinase; CG-CO_2_: Guanidine-CO_2_ functionalized chitosan; CK2: Casein kinase 2; c-Myc: v-Myc myelocytomatosis viral oncogene homolog; CO-Fe: Cobalt-ferric; CPP: Cell penetrating peptide; *DANCR*: Differentiation Antagonizing Non-Coding RNA; eEF2K: Eukaryotic Elongation Factor 2 Kinase; eGFP: Enhanced green fluorescent protein; EGFR: Epidermal growth factor receptors; FOXM1: Forkhead box protein M1; HB-EGF: Heparin-binding EGF like growth factor; ICAM-1: Intercellular Adhesion Molecule 1; lncRNAs: Long non-coding RNAs; LNP: Lipid nanoparticles; MDM2: Murine double minute; *MDR1*: Multidrug resistance gene 1; MPS1: monopolar spindle 1; MSNP: Mesoporous silica nanoparticles; mTORC2: Mammalian target of rapamycin complex 2; PAMAM: Poly (amidoamine) dendrimer; PEG: Polyethylene glycol; PEG_2000_-PE: Polyethyelene glycol2000-phosphatidyl ethanolamine; PEG-PLA: Poly(ethylene glycol)-b-poly(d,l-lactide); PEI: Polyethylimine; PIHCA: Polyisohexylcyanoacrylate; PLEGP: Poly[*bis*(ε-Lys-PEI)Glut-PEG]; PLK1: Polo-like kinase 1; PM: Polymeric micelles; PMLA: Poly(β-L-malic acid); RhoA: Ras homologous A; SWNTs: Single-walled carbon nanotubes; TBG: Tenfibgen; VEGF: Vascular endothelial growth factors; VEGFR: Vascular endothelial growth factor receptors.

**Table 3:** Anticancer siRNA-mediated nanoparticles in clinical trials.

Therapeutic Name	Indications	Target Gene/Protein	Route of Administration	Status	References
**Phase I**					
CALAA-01	Cancer, Solid tumor	RRM 2	Systemic/IV infusion	Terminated	[151]
ALN-VSP02	Solid Tumors	VEGF, KSP	Systemic/IV infusion	Completed	[152]
Mesenchymal Stromal Cells-derived iExosomes	Pancreatic Cancer	KRAS G12D Mutation	Systemic/IV infusion	Not yet recruited	[153]
siRNA-EphA2-DOPC	Advanced Cancers	EphA2	Systemic/IV infusion	Not completed yet	[154,155]
**Phase II**					
Atu027	Advanced or Metastatic Pancreatic Cancer (II), Solid Tumors (I)	PKN3	Systemic/IV infusion	Completed	[156,157,158]
TKM-PLK1 (TKM-080301)	Adrenal Cortical Carcinoma (II), Hepatocellular Carcinoma (II), Neuroendocrine Tumor (II), Solid Tumors (I)	*PLK1*	Systemic/IV infusion	Completed	[159,160,161]
siG12D LODER	Pancreatic Ductal Adenocarcinoma, Pancreatic Cancer	G12D mutated KRAS	Local/Surgical implantation	Ongoing	[162]
DCR-MYC	Solid Tumors, Hepatocellular Carcinoma, Multiple Myeloma, NonHodgkins Lymphoma, Pancreatic Neuroendocrine Tumors	MYC	Systemic/IV infusion	Terminated	[163]
**Phase III: No anticancer siRNA mediated nanoparticles have commenced yet**					

**Abbreviation:** DOPC: 1,2-dioleoyl-*sn*-glycero-3-phospahtidylcholine; i.v., intravenous; EphA2, EPH receptor A2 (ephrin type-A receptor 2); KSP, kinesin spindle protein; LNP, lipid nanoparticle; LODER, LOcal Drug EluteR; PKN3, protein kinase N3; PLK1, polo-like kinase 1; RRM2, ribonucleoside-diphosphate reductase subunit M2; siRNA: small interfering RNA; VEGF, vascular endothelial growth factor.
